# Early and unrestricted access to high-efficacy disease-modifying therapies: a consensus to optimize benefits for people living with multiple sclerosis

**DOI:** 10.1007/s00415-021-10836-8

**Published:** 2021-10-09

**Authors:** Massimo Filippi, Romano Danesi, Tobias Derfuss, Martin Duddy, Paolo Gallo, Ralf Gold, Eva Kubala Havrdová, Barbara Kornek, Francesco Saccà, Mar Tintoré, Jörg Weber, Maria Trojano

**Affiliations:** 1 Neurology Unit, Neurorehabilitation Unit, Neurophysiology Service, and Neuroimaging Research Unit, Division of Neuroscience, IRCCS San Raffaele Scientific Institute, Via Olgettina, 60, 20132 Milan, Italy; 2 Vita-Salute San Raffaele University, Milan, Italy; 3 University of Pisa, Pisa, Italy; 4 University of Basel, Basel, Switzerland; 5 The Newcastle Upon Tyne Hospitals, Newcastle upon Tyne, UK; 6 University of Padua, Padua, Italy; 7 Ruhr-Universität Bochum, Bochum, Germany; 8 Department of Neurology, First Medical Faculty, Charles University, Prague, Czech Republic; 9 Medical University Vienna, Vienna, Austria; 10 Università Degli Studi Di Napoli ‘Federico II’, Naples, Italy; 11 MS Centre of Catalonia at the Hospital Vall d’Hebron, Barcelona, Spain; 12 Klinikum Klagenfurt,, Klagenfurt am Wörthersee, Austria; 13 University of Bari, Bari, Italy

**Keywords:** Benefit–risk profile, Unrestricted access, Healthcare system, High-efficacy disease-modifying therapy, Multiple sclerosis, Pharmacoeconomics

## Abstract

Early intervention with high-efficacy disease-modifying therapy (HE DMT) may be the best strategy to delay irreversible neurological damage and progression of multiple sclerosis (MS). In European healthcare systems, however, patient access to HE DMTs in MS is often restricted to later stages of the disease due to restrictions in reimbursement despite broader regulatory labels. Although not every patient should be treated with HE DMTs at the initial stages of the disease, early and unrestricted access to HE DMTs with a positive benefit–risk profile and a reasonable value proposition will provide the freedom of choice for an appropriate treatment based on a shared decision between expert physicians and patients. This will further optimize outcomes and facilitate efficient resource allocation and sustainability in healthcare systems and society.

**Supplementary Information:**

The online version contains supplementary material available at 10.1007/s00415-021-10836-8.

## Introduction

Multiple sclerosis (MS), a complex chronic disease characterized by inflammation, neurodegeneration and inevitable progression, is the most common autoimmune disorder of the central nervous system (CNS) among young adults [1]. Approximately 85% of people living with MS (PLwMS) are diagnosed with relapsing MS (RMS), which includes relapsing–remitting MS (RRMS) that may later turn to secondary progressive MS [2]. MS may be seen as a dynamic continuum of phenotypic phases, with each phase being linked to a change in disability worsening that could result from poorly recovered relapses and progression [3].

According to the European Committee for Treatment and Research in Multiple Sclerosis (ECTRIMS) and European Academy of Neurology (EAN) guidelines, MS disease management aims to reduce the risk of relapses and potentially disability progression; however, no curative treatment is available to date [4]. Currently available disease-modifying therapies (DMTs) are commonly distinguished as moderate-efficacy (ME) DMTs (interferon-beta [IFNβ], glatiramer acetate, dimethyl fumarate, teriflunomide) and high-efficacy (HE) DMTs (alemtuzumab, cladribine, fingolimod, natalizumab, ocrelizumab, siponimod and the newly approved ozanimod and ofatumumab) [5].

**Fig. 1:**
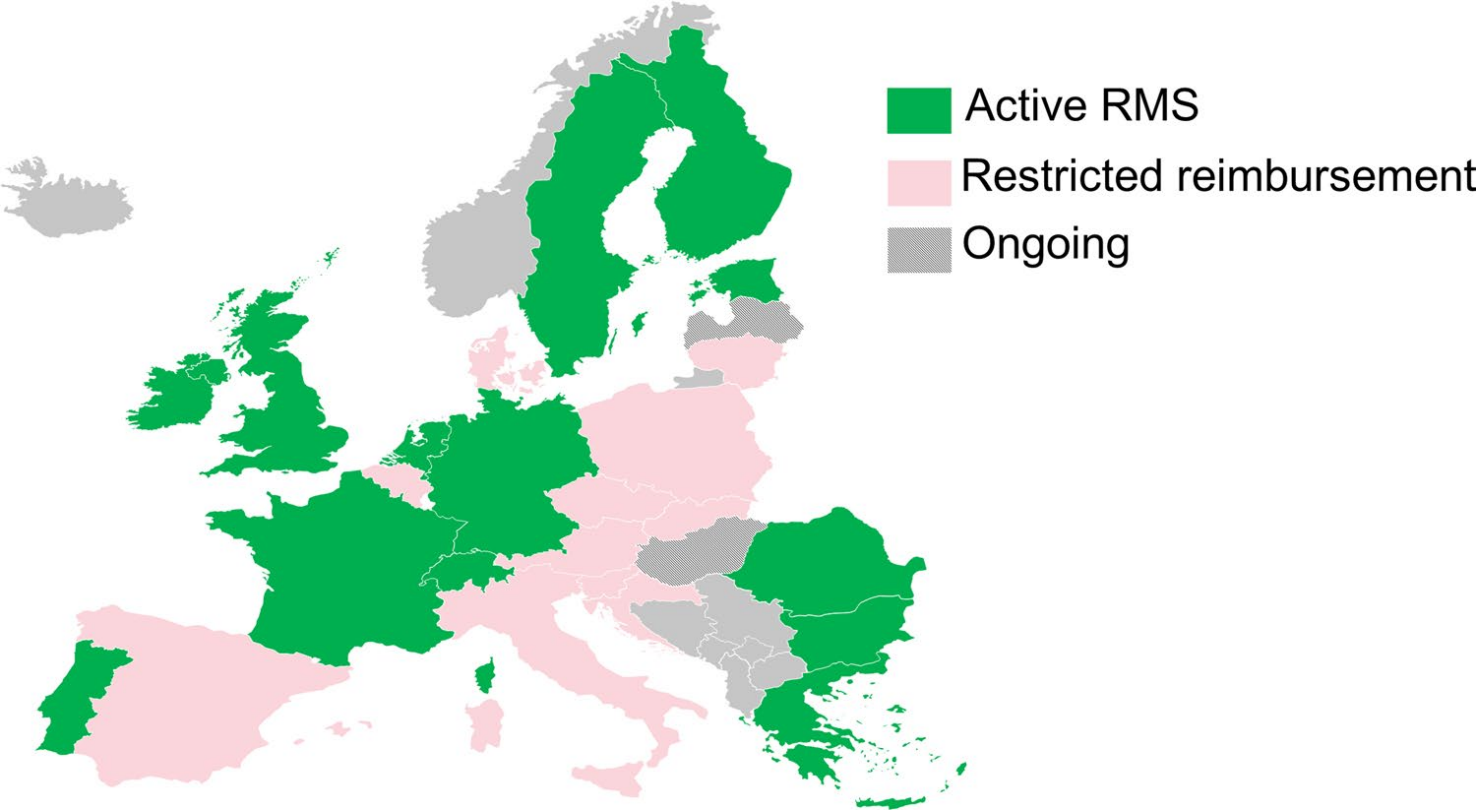
Reimbursement status for RMS for ocrelizumab compared with its EMA label (based on national reimbursement status; see supplementary material for the full list of sources). *EMA* European Medicines Agency, *RMS* relapsing multiple sclerosis

In European clinical practice, treatment choice is often influenced by limited access to HE DMTs for naïve patients, or those without highly active disease (defined by clinical relapses or MRI activity), due to restrictions on the approved regulatory label population imposed by reimbursement bodies [6]. Countries such as Italy and France require almost a year on average to complete the reimbursement process, which is linked to a lack of access to HE DMTs [7]. In Europe, approximately 20% of patients with MS get access to the most innovative treatments. Whereas, lesser proportions can be found in the eastern European countries (3–4%) [8]. This is the case for the recently developed HE DMT ocrelizumab, which, in spite of a broad European Medicines Agency (EMA) indication statement that renders it eligible for use in all RMS patients with active disease, has been restricted to a second or later line of treatment, except in highly active naïve patients **(**Fig. 1), by certain European reimbursement authorities such as those in Spain and Italy (Online Resource 1) [9]. Such restrictions impose a line-based escalation treatment approach, i.e., start with a low-risk, ME immunotherapy, and switch to more efficacious therapies if breakthrough disease activity is encountered [6]. Similarly, in most European countries, as per EMA label, it is recommended to have natalizumab be fully reimbursed. However, this is not the case in all countries, for example, Italy and the Czech Republic had imposed restrictions on patients who have failed to respond to a full and adequate course (normally at least one year of treatment) of IFNβ. Belgium has further restrictions for patients with EDSS ≤ 6.5, whilst countries have later broadened their patient group to also include patients who failed under other 1st line therapies. Other countries such as Austria, Netherlands, Slovakia have opted for only reimbursing for patients who have failed to respond to a full and adequate course of 1st line therapy [10]. The limited accessibility of HE DMTs in some European countries might have an impact on their overall utilization rate. In Europe, in 2019, only 23% of PLwMS received HE DMTs as first-line treatment, with only 7% of PLwMS on monoclonal antibody treatment despite the availability of monoclonal antibody therapies for MS since 2009. HE DMT utilization as second- and third-line treatment increased to 58% and 75%, respectively, highlighting a general need to switch and escalate to a HE DMT for the majority of PLwMS over the course of the disease [11].

In a chronic progressive disease such as MS, where time is of the essence for treatment, limiting early access to reimbursed HE DMTs may result in a lost therapeutic opportunity [12] and restriction in choice of an appropriate therapy by physicians. In contrast, unrestricted and early access to HE DMTs would enable physicians to tailor treatment choice based on individual patient characteristics that go beyond the current highly active versus non-highly active disease classification, which is of great importance given the high heterogeneity of MS and is in line with the ECTRIMS/EAN guidelines [4]. Accordingly, PLwMS without highly active disease at onset could benefit from HE DMTs during the critical early stages of the disease.

According to the latest European Public Assessment Report (EPAR), the uncertainties regarding the imbalance in malignancies observed in the ocrelizumab trial were stated as the rationale for restrictions [13]. Pharmacoeconomic and budget impact considerations may also have caused further access restrictions. Here, we aim to outline the importance of providing early and unrestricted access to HE DMTs with a positive benefit–risk profile, to optimize patient outcomes and reduce the direct, indirect and societal costs related to MS for healthcare systems (HCS), which are dependent on responsible pricing strategies that align with unrestricted access. We present both evidence and suggestions based on professional experiences from clinical healthcare professionals and payer advisors as discussed during an expert meeting in January 2021.

## Early use of HE DMTs can improve PLwMS outcomes

**Fig. 2:**
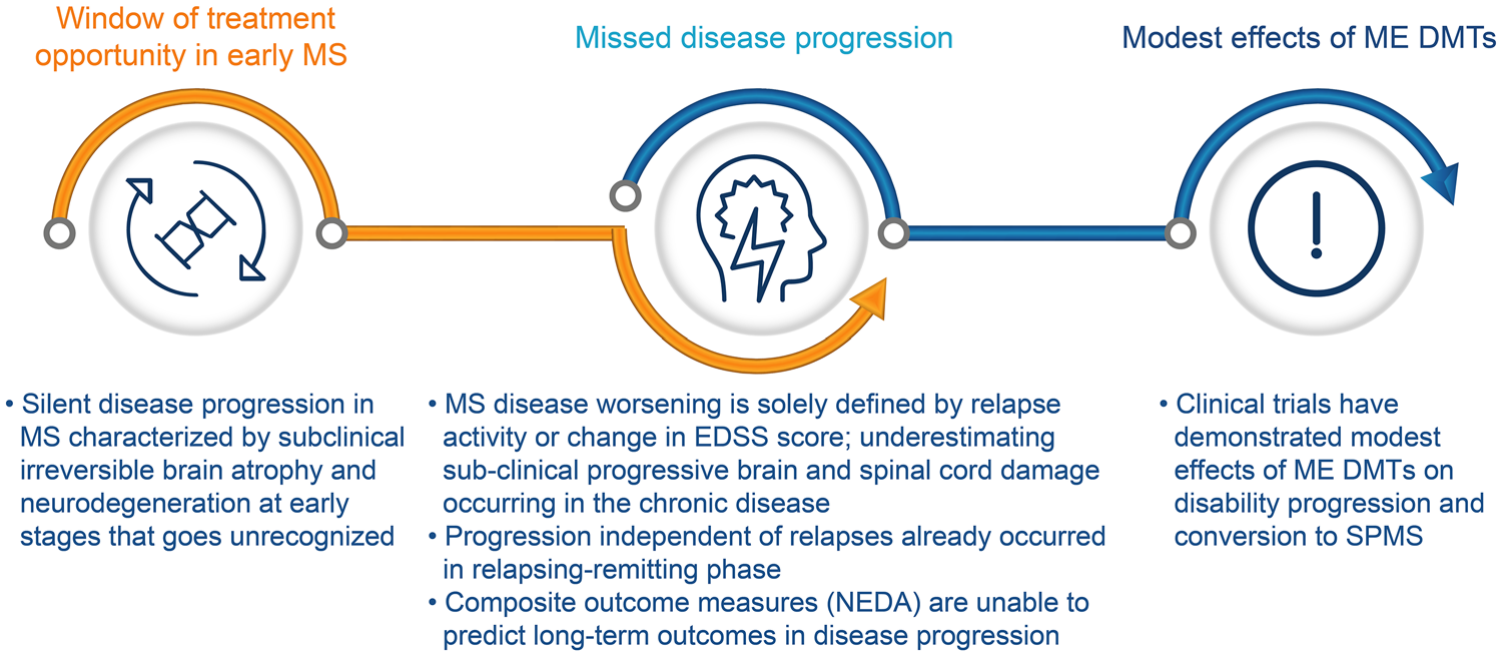
Different factors highlighting the need for early initiation of HE DMTs in PLwMS. *EDSS* Expanded Disability Status Scale, *DMT* disease-modifying therapy, *HE* high efficacy, *ME* moderate efficacy, *MS* multiple sclerosis, *NEDA* no evidence of disease activity, *PLwMS* people living with MS, *SPMS* secondary progressive MS

The increasing understanding of the natural history and heterogeneous nature of MS, paired with growing evidence suggesting improved patient outcomes with early initiation of HE DMTs, questions the current escalation strategy and calls for a change in the MS management. Several factors highlight the need for early initiation of HE DMTs (Fig. 2) [3, 5, 6, 12, 14, 15].

The timing of HE DMT has a long-term impact on the neurological impairment of patients [16, 17]. Several studies suggest that maximum benefit can be achieved with early initiation of HE DMTs, even in the absence of poor prognostic factors at the initial diagnosis. A recent study which compared the patients who started with HE DMT and that of who started ME DMT as the initial therapy and found a decreased risk of 6-month confirmed EDSS deterioration and a lower probability of on-treatment relapses [18]. The HE DMTs such as fingolimod and natalizumab take less time to restore the immune system hence they could be potential candidates for early sequencing [19]. The HE DMTs alemtuzumab, fingolimod, ocrelizumab, and ofatumumab have demonstrated improved efficacy across several clinical programs versus interferons or teriflunomide in reducing the relapse rate and/or delaying disease progression, as evidenced by MRI lesions and brain volume loss [20–23]. Studies demonstrate favorable short- and long-term outcomes with regard to relapse activity and disability worsening following early versus late initiation of an HE DMT (Table 1) [24–27].

**Table 1:** Studies demonstrating favorable outcomes with early initiation of HE DMTs in PLwMS [23–26]

Study	Study details	Outcomes
He et al. [16]	Retrospective data from the MSBase registry and the Swedish MS registry	Compared long-term disability outcomes of PLwMS starting HE DMTs within two years of disease onset vs those starting only 4–6 years after disease onset
		Study reported lower EDSS progression of PLwMS on early HE treatment after 6–10 years of follow-up, amounting to an adjusted mean difference in EDSS score between groups over the whole follow-up period of -0.98 points
Harding et al. [24]	Single-center study on population-based cohort of PLwMS in southeast Wales	Classified data according to first-line treatment strategy into early intensive vs escalation strategy
		Study reported more favorable long-term outcomes, measured by EDSS, following early intensive therapy vs escalation therapy
Brown et al. [23]	Prospective cohort study utilizing propensity score matched data from 68 neurology centers in 21 countries	Study highlights the risk of disease progression with later initiation of HE DMTs, associating a lower risk of RRMS to SPMS conversion in PLwMS receiving an initial HE DMT (fingolimod, alemtuzumab or natalizumab) vs glatiramer acetate or interferon
Uher et al. [26]	Longitudinal study of patients with RRMS	Study demonstrated efficacy of HE DMTs to decelerate progression, as characterized by brain volume loss
		Effects were only measurable two years after escalation to a HE DMT, again highlighting the time lost in case of treatment escalation

*EDSS* Expanded Disability Status Scale, *DMT* disease-modifying therapy, *HE* high efficacy, *ME* moderate efficacy, *MS* multiple sclerosis, *PLwMS* people living with MS, *RRMS* relapsing–remitting MS, *SPMS* secondary progressive MS

Moreover, the impact of age on treatment efficacy must be considered when defining a treatment strategy. One shortcoming of current clinical trial data is the lack of trials enrolling a significant number of patients aged > 55 years, challenging efforts to draw definitive conclusions thereon. A recent meta-analysis of MS clinical trials indicated that age is an essential modifier of a drug’s efficacy. More specifically, the results suggest that HE DMTs have greater efficacy than ME DMTs in younger patients aged ≤ 40.5 years, after which this added benefit is lost for the average patient, emphasizing the importance of leveraging the early window of opportunity with HE DMTs to delay disease progression [28].

Taken together, early and unrestricted access to HE DMTs will allow physicians and patients to jointly decide on the optimal treatment strategy and can likely offer the best strategy to diminish irreversible neurological damage and progression, which cannot be achieved when initiated at already progressed disease stages [9]. Moreover, a recent cohort study showed a 29% reduction in disability progression in the Swedish MS patients from a national registry, where HE DMTs were used from the beginning of the disease treatment, as compared to Danish patients where an escalation treatment strategy was employed [29]. Further research is ongoing, investigating the benefits of early use of HE DMTs in clinical trials, which will provide controlled and randomized data in the near future [30, 31].

## Improved benefit–risk profile of novel HE DMTs warrants their early use

Before 2010, safety concerns were the major factors impacting the decision for late-stage utilization of HE DMTs. These include an increased risk of developing progressive multifocal leukoencephalopathy with natalizumab [5], a high rate of secondary autoimmune disease with alemtuzumab [5, 21], an increased risk of infections, malignancies, cardiovascular effects, and macular edema with fingolimod [19, 20, 32], and a reduction in lymphocyte counts with cladribine [33].

The two anti-CD20 antibodies ofatumumab and ocrelizumab showed a favorable short-term safety profile, which was comparable to the ME control treatments used in their respective clinical trials (vs teriflunomide and INFβ-1a, respectively); therefore, mitigating safety concerns is a key reason for second-line usage of these HE DMTs. The main adverse events included mild or manageable injection-related reactions, although these were not of major concern [22, 23]. In addition, an imbalance in neoplasms was observed with ocrelizumab; however, the incidence was within the background rate expected for an MS population [7, 23].

Furthermore, for the novel HE DMTs ocrelizumab and ofatumumab, there is still a need to differentiate between short- and long-term safety concerns, with the latter requiring more clinical and real-world data to be adequately assessed. In addition, the improved safety profile, optimized pharmacokinetic/pharmacodynamic properties, and corresponding improved risk/benefit profile of novel anti-CD20s warrant their early use without the need for trade-offs [22, 23, 34].

While long-term safety data of novel HE DMTs are still outstanding, it is important to put the relative risks associated with early use of HE DMTs in perspective with the risks and outcomes of an alternative treatment approach based on treatment escalation. The misperception of risk might result in treatment inertia and resistance by the treating physician to transition from an established to a novel treatment strategy, which is difficult to tackle outside of centers of excellence, where neurologists lack the experience and comfort to use HE DMTs early. Therefore, the risk/benefit ratio for each treatment approach must be carefully assessed to counterbalance the subjective risk perception in consideration of granting early and unrestricted access to such drugs.

The long-term risk–benefit ratio of HE DMTs is likely more favorable when initiated at a younger age. While the more active pro-inflammatory immune system of younger PLwMS might proportionally yield a greater therapeutic effect, the profound weakening of the immune system increases the risk of infections and cancer associated with immunomodulatory DMTs in elderly PLwMS [28, 35]. Different wash-out periods and the risk of infections associated with prior/sequential immunosuppression must be considered during treatment decision-making. As initial treatment switches are often caused by poor efficacy of the first-line drug used, an early HE treatment strategy likely reduces the associated risks [12, 36]. Moreover, the potential for a marginally increased long-term risk of infections with an HE versus ME DMT strategy should be balanced with the evidence of accelerated disability progression associated with the latter, more conservative treatment strategy, which neglects the early window of therapeutic opportunity to prevent irreversible brain damage. From a patient perspective, such a conservative strategy would result in a reduction in the quality of life (QoL) and hence a potentially reduced working capacity at a younger age, which are important factors to be considered early in the course of the disease.

In conclusion, the positive benefit/risk ratio of novel HE DMTs should suffice to allow for their early use, providing PLwMS the chance to immediately benefit from newly available HE treatments to maximally improve their long-term outcomes, which is of utmost importance. It is still paramount for novel therapies to collect long-term safety data to generate greater certainty on their benefit/risk profile over time, however, it should not be the underlying reason to restrict access and delay potential improved outcomes in PLwMS.

## Long-term pharmaco-economic assessments may demonstrate societal benefits of early and unrestricted access to HE DMTs

The overall societal cost of MS, including direct medical, non-medical and indirect costs, amounts to €15.5 billion/year in Europe [6]. Usually, the direct non-medical costs, which account for two-thirds of the overall cost, fall outside the HCS budgets and are borne by PLwMS and their families [6]. Therefore, a thorough assessment of the economic benefits of MS treatments should include long-term clinical and health-related QoL outcomes and all societal costs [37].

Primarily, this would entail taking into consideration the impact of treatment efficacy and early intervention on delaying disability progression and the associated influence on the QoL and productivity of PLwMS [38], which is the main driver of MS societal costs [6]. International surveys demonstrated a relationship between the level of efficacy of the DMT used and improved QoL and productivity [39].

In addition, HCS costs may be reduced using oral and self-administered HE DMTs, which can reduce hospital occupation and the risk of infection, a problem magnified by the current coronavirus disease 2019 (COVID-19) pandemic [40].

The long-term cost-effectiveness of early versus late use of HE DMTs has been studied in numerous analyses, most of which confirm the positive socio-economic impact of early use of HE DMTs [41]. It is suggested that initial investment in the early use of HE DMTs may reduce overall costs in the long-term by reducing disability progression [41]. However, such long-term benefits and savings might be at odds with payers’ short-term budget needs, and, additionally, overall societal costs may be beyond canonical payers’ remit.

Comprehensive cost-consequence analyses can provide data on different benefits of treatments to HCS and society that are relevant for payers to inform their decision-making at a national, regional and hospital level. From a budget impact perspective, the availability of HE DMTs with a positive risk/benefit profile and a reasonable price proposition allows for their use early in the course of the disease, which would positively impact affordability, HCS sustainability and cost savings.

## Discussion/future directions

Early and unrestricted access to HE DMTs with a positive benefit–risk profile would provide freedom of choice of an appropriate therapy by expert physicians, optimize PLwMS outcomes and reduce HCS and societal costs. As evidenced by clinical trials and retrospective studies, the use of HE DMTs is clinically relevant to leverage the early window of therapeutic opportunity, improve outcomes and delay disability progression in PLwMS. The overall safety profile of novel HE DMTs is comparable to other DMTs currently being used as first-line therapies based on head-to-head clinical trial data. Even though we acknowledge the need for long-term, real-world safety data, this should not be the reason to restrict access to novel HE DMTs, as this would potentially translate to 5- to 10-year delayed access. Moreover, novel HE DMTs may positively impact overall cost-effectiveness and HCS sustainability and even result in savings when all direct and indirect costs are holistically assessed over the long term, particularly with a reasonable pricing strategy.

However, to accelerate the transition towards unrestricted access to HE DMTs, some changes might be required from a payer, HCS and societal perspective, as well as from the pharmaceutical industry and in MS clinical practice.

Overall, there is a need to improve awareness in political decision-makers about the burden of the disease and its associated societal cost. Only then can the socio-economic benefits linked to early use of HE DMTs and relative savings for the society, which stretch beyond the short-term budget interest of traditional payers, be contextualized and considered when assessing the opportunity to invest in early use of HE DMTs.

Additionally, a change in payers’ perception regarding risk assessment and risk trade-offs, in line with the clinical consensus on the benefits of HE DMTs for PLwMS, needs to be fostered. This might be achieved by facilitating communication, for example, through exchange platforms, between payers and/or clinical specialists of countries with differing access restrictions to HE DMTs.

Furthermore, unrestricted access to HE DMTs will need to be sustainably adopted in clinical practice by neurologists, beyond the main medical experts and centers of excellence. Peer-to-peer events that increase neurologists’ awareness of the benefits associated with such an approach, and their comfort implementing it, may accelerate adoption and support overcoming treatment inertia commonly associated with novel treatment strategies.

As an intermediate step towards sustainable unrestricted and early access to HE DMTs, decisions in clinical practice might be favored through adoption of a more comprehensive therapeutic algorithm than that currently used based on heterogeneous definitions of disease course/activity classifications. Such an algorithm could encompass the integration of a range of prognostic factors to stratify PLwMS in high- and low-risk groups, thereby determining their eligibility for HE DMT treatment [42]. Therefore, in addition to supporting clinical decision-making, a novel therapeutic algorithm to determine HE DMT eligibility based on integrated prognostic factors can provide additional budget certainty to payers mitigating their concerns of an uncontrolled budget expenditure if unrestricted access to HE DMTs were to be granted indiscriminately to all PLwMS.

Prognostic markers may include demographic and environmental factors, as well as clinical and radiological characteristics [5, 42]. Nevertheless, it must be acknowledged that, to date, the disease course cannot be exactly predicted and further studies would be necessary to validate such a therapeutic algorithm. In the future, progressive improvements in predicting long-term outcomes will enable a more tailored treatment of MS, which will likely result in a more efficient use of HCS resources.

In addition to prognostic markers the importance of shared decision-making between physicians and patients in determining treatment choice is commonly accepted [4, 5, 43]. Taking into account patient preferences, such as side effects, mode and frequency of administration and intensity of monitoring, is considered an important component of care for chronic diseases, which can improve acceptance of and adherence to DMTs. Thus, an optimal therapeutic approach for PLwMS should allow a certain flexibility to adjust for patient preferences and individual patient characteristics, especially considering the ongoing COVID-19 pandemic [43].

## Supplementary Information

Below is the link to the electronic supplementary material.

Supplementary file1 (PDF 87 KB) Online Resource 1 Sources of national reimbursement status of ocrelizumab in Europe

## Data Availability

Not applicable.
