# Supplementary material for: Early and unrestricted access to high-efficacy disease-modifying therapies: a consensus to optimize benefits for people living with multiple sclerosis
Source: J Neurol. 2021 Oct 9;269(3):1670–7. doi: 10.1007/s00415-021-10836-8 (PMC8501364; doi:10.1007/s00415-021-10836-8)
Supplement: Supplementary file 1 — Supplementary file1 (PDF 87 KB) Online Resource 1 Sources of national reimbursement status of ocrelizumab in Europe [file 415_2021_10836_MOESM1_ESM.pdf]

COUNTRY	SOURCE / Link to document
Austria	https://www.sozialversicherung.at/oeko/views/detail.xhtml?pharmaNumber=4472121
ROMANIA	https://tinyurl.com/yxckfvbv
Netherlands	https://www.medicijnkosten.nl/medicijn?artikel=OCREVUS+INFVLST+CONC+300MG%2F10ML+%2830MG%2FML%29+FLACON&id=79fbfd3b3ee4c175ecc222ee4d400380
France	https://www.has-sante.fr/upload/docs/evamed/CT-16878_OCREVUS_PICins_SEP-R_avis2_CT16878_def.pdf<br>https://www.legifrance.gouv.fr/jorf/id/JORFTEXT000038175160?tab_selection=all&searchField=ALL&query=OCREVUS&page=1&init=true<br>https://www.has-sante.fr/upload/docs/application/pdf/2018-07/ocrevus_pic_ins_avis_3_ct16833.pdf<br>https://www.legifrance.gouv.fr/jorf/id/JORFTEXT000038175207?tab_selection=all&searchField=ALL&query=OCREVUS&page=1&init=true
Lithuania	Reimbursed medicines list with reimbursed indications – link.<br>MS treatment guidelines – link.
Portugal	https://www.infarmed.pt/documents/15786/1424140/Relat%C3%B3rio+p%C3%BAblico+de+avalia%C3%A7%C3%A3o+de+Medicamento+Ocrevus+%28DCI+ocrelizumab%29+2020/5f7e7157-561c-6e22-5582-199404eedb1f
Italy	https://www.gazzettaufficiale.it/eli/gu/2018/09/03/204/sg/pdf<br>Registry:<br>https://www.gazzettaufficiale.it/do/atto/serie_generale/caricaPdf?cdimg=18A0571300100010110001&dgu=2018-09-03&art.dataPubblicazioneGazzetta=2018-09-03&art.codiceRedazionale=18A05713&art.num=1&art.tiposerie=SG
Slovenia	http://www.cbz.si/cbz/bazazdr2.nsf/o/4E6B82372BD70082C125822700050445?opendocument
Poland	https://www.gov.pl/web/zdrowie/obwieszczenie-ministra-zdrowia-z-dnia-21-pazdziernika-2020-r-w-sprawie-wykazu-refundowanych-lekow-srodkow-spozywczych-specjalnego-przeznaczenia-zywieniowego-oraz-wyrobow-medycznych-na-1-listopada-2020-r
Sweden	https://janusinfo.se/nationelltinforandeavlakemedel/produktinfo/ocrevusokrelizumab.4.1dfa69ad1630328ad7c3b9c3.html<br>https://www.tlv.se/download/18.15b37133165bb795253817be/1536757705126/bes180903_underlag_ocrevus.pdf
Slovakia	https://www.health.gov.sk/Clanok?lieky202011 (section - Časť B: Indikačné obmedzenia)
Croatia	http://www.hzzo.hr/zdravstveni-sustav-rh/trazilica-za-lijekove-s-vazecih-lista/
Czech Republic	http://www.sukl.cz/modules/medication/detail.php?code=0222682&tab=prices
GERMANY	https://www.iqwig.de/en/projects/a18-06.html
Luxembourg	https://cns.public.lu/dam-assets/legislations/texte-coordonne/med-comm/2011-liste-comm.pdf
Bulgaria	https://www.nhif.bg/page/45
Finland	
Estonia	https://www.riigiteataja.ee/akt/118082020001
Switzerland	http://www.spezialitätenliste.ch
Hungary	http://neak.gov.hu//data/cms1027540/PUPHA_GYOGYSZER_LAKOSSAGI_20201101_v3.xls<br>This excel contains all the reimbursed therapies, there is no Ocrevus in it.
Latvia	http://www.vmnvd.gov.lv/lv/kompensejamie-medikamenti/kompensejamo-zalu-saraksti
Denmark	Link
Spain	https://www.mscbs.gob.es/profesionales/medicamentos.do?metodo=verDetalle&cn=720456 (MoH database)<br>https://www.aemps.gob.es/medicamentosUsoHumano/informesPublicos/docs/IPT-ocrelizumab-Ocrevus-esclerosis-multiple.pdf?x53593 (HTA report)
